# Lactate regulates cell differentiation of erythroid progenitor cells via histone lactylation modification

**DOI:** 10.1016/j.isci.2025.112842

**Published:** 2025-06-09

**Authors:** Qianqian Yang, Hengchao Zhang, Yan Hou, Shaoyang Gu, Lixiang Chen, Fumin Xue, Xiuyun Wu

**Affiliations:** 1 School of Life Sciences, Zhengzhou University, Zhengzhou 450001, China; 2 Department of Gastroenterology, Children’s Hospital affiliated of Zhengzhou University, Zhengzhou 450000, China

**Keywords:** Biochemistry, Molecular biology, Cell biology

## Abstract

Lactate plays important regulatory roles in a variety of biological events by regulating metabolic homeostasis and histone lactylation. However, the role of lactate and histone lactylation in human erythropoiesis remains unclear. Here, we explored the role of lactate in erythropoiesis by adding the glycolysis inhibitor 2-deoxy-*d*-glucose (2-DG) or exogenous lactate Na-La to decrease or increase intracellular lactate levels. The results showed the inhibition of glycolysis promoted erythroid progenitors’ differentiation, blocked cell cycle, and reduced colony formation of colony-forming unit-erythroid (CFU-E), whereas elevated lactate levels delayed erythroid progenitors’ differentiation and promoted CFU-E colony formation. We also found lactate levels directly regulated H3K14la intensity. Furthermore, we showed changes in H3K14la abundance and gene expression of the cell cycle and division-related genes *CCNB1*, *CFL1*, *CENPA*, and *GNAI2*, which were associated with stem cell pluripotency and differentiation. In conclusion, our study reveals lactate affects cell differentiation of early erythroid progenitors by regulating gene expression through histone lactylation.

## Highlights

• Altered lactate levels regulate erythroid progenitor cell differentiation
• Lactate levels directly regulated H3K14la abundance
• 2-DG treatment regulates the expression of cell cycle and division-related genes
• Expression of cell cycle and division-related genes related to H3K14la abundance

## Graphical abstract

**Figure:**
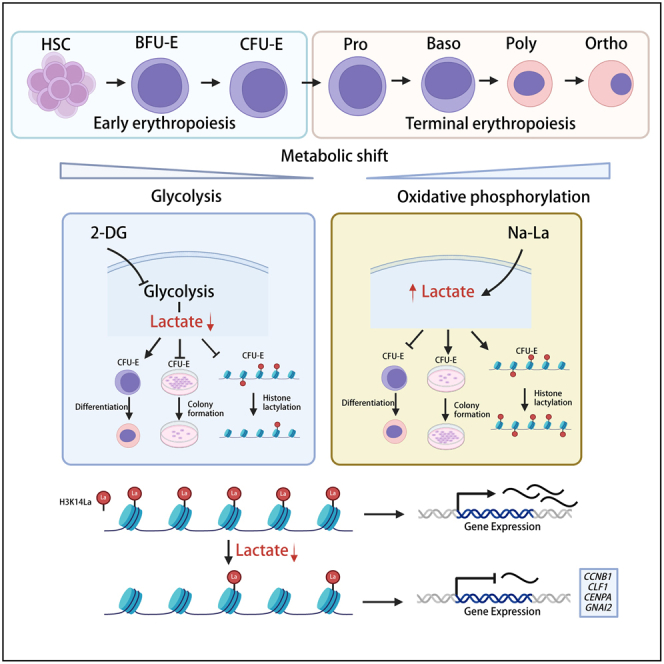

## Introduction

Erythropoiesis is the process by which hematopoietic stem cells (HSCs) proliferate and differentiate to produce mature red blood cells. It is a tightly regulated process that can be divided into 3 stages: early erythropoiesis, terminal erythroid differentiation, and reticulocyte maturation.^1^^,^^2^ During the early stage of erythropoiesis, HSCs sequentially give rise to common myeloid progenitor, megakaryocyte-erythrocyte progenitor, burst-forming unit-erythroid (BFU-E), and colony-forming unit-erythroid (CFU-E) cells. BFU-E and CFU-E cells have been traditionally defined by colony assays. Erythroid progenitors possess the capacity for self-renewal.^3^ During the stage of terminal erythroid differentiation, the glycoprotein GPA is an essential factor for the erythroid progenitor cells to differentiate into terminal erythroid cells. Morphologically recognizable proerythroblasts (Pro-E) undergo mitosis to produce basophilic erythroblasts, polychromatic erythroblasts (Poly-E), and orthochromatic erythroblasts (Ortho-E). Ortho-E expel their nuclei to generate reticulocytes. Finally, reticulocytes mature into red blood cells, initially in bone marrow and then in the circulation.^3^^,^^4^^,^^5^

Erythropoiesis is synergistically regulated by a variety of factors in which cytokines, transcription factors, cell-cell contact, and metabolism play important roles.^6^^,^^7^^,^^8^^,^^9^ Several metabolic activities such as glucose metabolism, lipid metabolism, hemoglobin metabolism, and various metabolites contribute to the maintenance of cellular activities and the regulation of erythroid differentiation.^10^^,^^11^ Among them, glycolysis not only provides mature erythrocytes with the energy required for cellular activities but also participates in the regulation of HSCs self-renewal, terminal erythroid differentiation, and enucleation.^6^^,^^10^^,^^12^ A shift in the metabolic state from glycolysis to oxidative phosphorylation occurs as HSCs exit the quiescent state and differentiate into erythroid lineage cells, and suppression of glycolysis stimulates erythroid lineage commitment.^13^^,^^14^

Besides producing energy for proliferation, survival, and differentiation of living cells, metabolites serve as reactants for a variety of biomolecular synthesis pathways and substrates for epigenetic modifications.^15^^,^^16^ Recent research has found that lactate is an important product of glycolytic metabolic processes; it can not only participate in the regulation of various cellular activities through metabolic processes but also act as a modification substrate to participate in histone lysine lactylation (Kla), thereby activating the transcription of downstream genes and participating in the regulation of important cellular functions.^17^^,^^18^ The level of lactate leads to an increase in Kac (H3K27ac) and Kla (H3K18la) of histones in the promoter region of pluripotency factor genes *OCT4*, *SOX2*, *KLF4*, and *MYC*, promoting the transcription of pluripotency factor genes and promoting somatic cell reprogramming.^19^ Glycolysis is the main energy supply for early erythropoiesis; although inhibition of glycolysis enhances erythroid differentiation, the mechanism is not fully understood. Moreover, lactate, one of the metabolites of glycolysis, in erythropoiesis are also unknown.

In this study, glycolysis inhibition using 2-deoxy-*d*-glucose (2-DG) resulted in decreased intracellular lactate levels, inhibited proliferation, impaired colony formation ability, and accelerated differentiation of erythroid progenitor cells. Adding exogenous lactate resulted in enhanced colony formation ability and arrested differentiation of erythroid progenitor cells. Moreover, 2-DG treatment resulted in reduced H3K14la and H3K18la abundance in erythroid progenitor cells, whereas they were elevated by addition of exogenous lactate. Mechanistically, 2-DG treatment caused a decrease in the cell cycle-related gene expression and H3K14la abundance in the promoter region. In conclusion, we demonstrated that lactate, one of the glycolytic metabolites, affects self-renewal and differentiation of erythroid progenitor cells. Also, lactate-mediated histone modifications of H3K14la play an important role in early stages of erythroid differentiation.

## Result

### Inhibition of glycolysis leads to impaired cell proliferation and arrested cell cycle during early erythropoiesis

**Figure 1:**
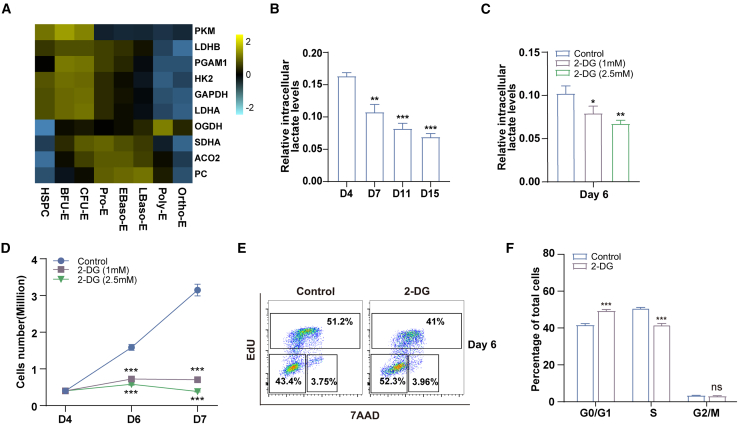
Inhibition of glycolysis inhibited cell proliferation and arrested cell cycle during early stage of human erythropoiesis (A) Heatmap analysis of the expression of genes involved in glycolysis and oxidative phosphorylation by RNA-seq during each erythroblast stage, cultured from normal human. (B) Colourimetric detection of intracellular lactate levels prepared from erythroblasts cultured for 4, 7, 11, and 15 days. Quantitative analysis of intracellular lactate level from 3 independent experiments. Cells were washed extensively to exclude extracellular lactate prior to lysis. (C) Quantitative analysis of intracellular lactate level in cells cultured for 6 days, including DMSO control, 2-DG (1 mM), and 2-DG (2.5 mM). (D) Growth curves of cells cultured for 4, 6, and 7 days, including DMSO control, 2-DG (1 mM), and 2-DG (2.5 mM). (E) Representative flow cytometry profiles of the cell cycle as assessed by EdU and 7-AAD staining of day 6 erythroid cells. (F) Quantitative analysis of the cell cycle from 3 independent experiments. Statistical analysis is from 3 independent experiments, and the bar plot represents mean ± SD of triplicate samples. ns, not significant; ∗*p* < 0.05, ∗∗*p* < 0.01, and ∗∗∗*p* < 0.001.

To confirm the metabolic switch from glycolysis to oxidative phosphorylation during erythropoiesis, we first analyze the expression levels of enzymes that related to glycolysis and oxidative phosphorylation from the transcriptomics data of highly purified populations of erythroid cells from cord blood at distinct stages of erythropoiesis.^20^^,^^21^ The gene expression of glycolysis-related enzymes (Hexokinase 2 [HK2], Glyceraldehyde-3-Phosphate Dehydrogenase [GAPDH], Phosphoglycerate Mutase 1 [PGAM1], Pyruvate Kinase M1/2 [PKM], Lactate Dehydrogenase A [LDHA], and Lactate Dehydrogenase B [LDHB]) was decreased, whereas gene expression of oxidative phosphorylation-related enzymes (Aconitase 2 [ACO2], Succinate Dehydrogenase Complex Flavoprotein Subunit A [SDHA], and Pyruvate Carboxylase [PC]) was initially increased (Pro-E) and then decreased (from Early Baso-E to Ortho-E) during erythropoiesis (Figure 1A). Interestingly, gene expression of the oxidative phosphorylation-related enzyme Oxoglutarate Dehydrogenase (OGDH) was consistently increased from early to late erythropoiesis (from Hematopoietic Stem/Progenitor Cell [HSPC] to Poly-E). Then we determined glycolysis and Oxidative phosphorylation (OXPHOS) during erythroid differentiation, and results similarly confirm this conclusion (Figures S1A–S1C). The active glycolysis process exhibited in erythroid progenitors may led to the production of substantial amounts of lactate, and we then measure lactate levels during erythropoiesis (Figure 1B). The results show that lactate levels dramatically decrease during erythropoiesis, which further support this metabolic shift. To investigate the effect of glycolysis on erythroid progenitors, we added the glycolysis inhibitor 2-DG on day 4 of erythroid differentiation and detected the intracellular lactate level on day 6. The results showed that 2-DG significantly reduced intracellular lactate levels by ∼22.3% and ∼34.2% in a dose-dependent manner (Figure 1C). Furthermore, treatment with 2-DG significantly inhibited the proliferation of erythroid progenitor cells (Figure 1D). Subsequently, we assessed apoptosis in early erythroid cells using annexin V^+^ and 7-AAD staining. The representative flow cytometry results are shown in Figure S2A. Quantitative analysis showed no significant difference in the cell apoptosis rate between control and 2-DG treatment groups (∼2.65%, ∼3.84%, and ∼4.87%; Figure S2B). To investigate the reasons for the inhibition of cell proliferation, we performed flow cytometry to examine the cell cycle using a 5-ethynyl-2′-deoxyuridine (EdU) incorporation assay. Cell cycle assays showed that inhibition of glycolysis led to an increase in early erythroid cells in the G0/G1 phase and a decrease in those in the S phase treatment with 2-DG (Figures 1E and 1F).

Lactate is an important product derived from glycolysis and has been shown to be implicated in tumor metabolism, as well as coordination signaling among various cellular entities, organs, and tissues.^22^^,^^23^ To investigate whether changes in lactate level constitute one of the pivotal factors mediating the influence of glycolysis on the development of early erythroid progenitor cells, exogenous lactate was added on day 4 during erythroid differentiation followed by assessment intracellular lactate levels on day 6. The results showed that addition exogenous lactate significantly increased intracellular lactate levels (Figure S2C). We used 10 and 25 mM sodium L-lactate (Na-La), respectively, to treat erythroid progenitor cells on day 4 and detected early erythroid differentiation on day 6. In addition, treatment with Na-La had no significant effect on the proliferation and apoptosis of erythroid progenitors (Figures S2D–S2F). Elevated intracellular lactate levels had no significant effect on the cell cycle (Figures S2G and S2H).^24^ This suggests that inhibition of glycolysis, but not lactate, leads to impaired proliferation and arrested cell cycle progression for early erythropoiesis.

### Lactate levels affect erythroid progenitor differentiation and colony formation

**Figure 2:**
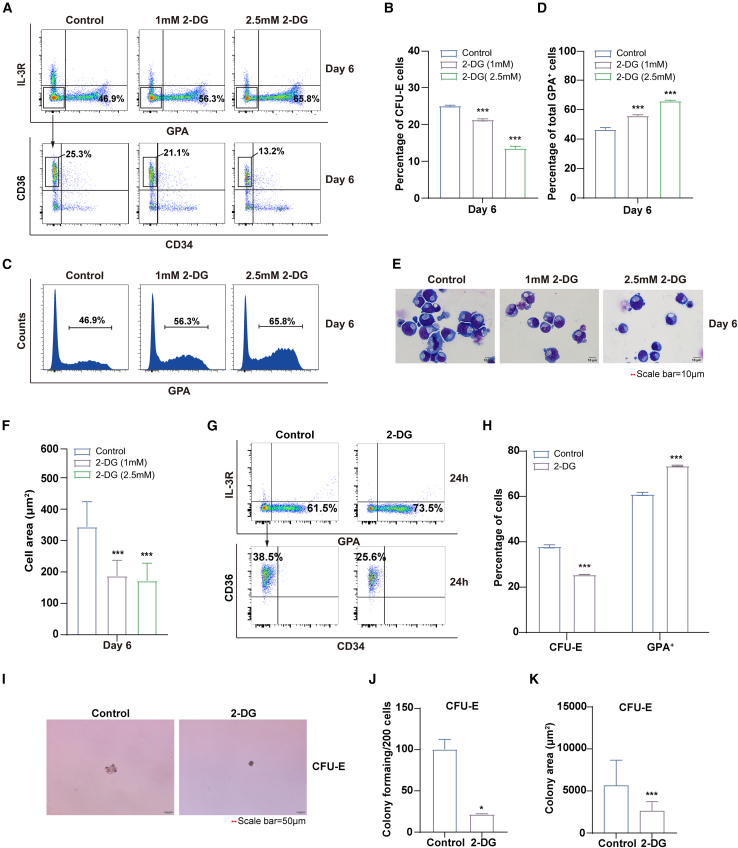
Inhibition of glycolysis promoted erythroid progenitor differentiation and colony formation (A) Flow cytometry analysis of erythroid progenitor cells at day 6, including DMSO control, 2-DG (1 mM), and 2-DG (2.5 mM). (B) Statistical results of the percentage of CFU-E cells to total cell. (C) Flow cytometry analysis of GPA in erythroid cells cultured on day 6, including DMSO control, 2-DG (1 mM), and 2-DG (2.5 mM). (D) Statistical results of the percentage of the GPA^+^ cells to total cell. (E) Representative images of DMSO control, 2-DG (1 mM), and 2-DG (2.5 mM) on day 6. (F) Quantitative analysis of cell area of day 6 erythroid cells (*n* = 100) from 3 independent experiments, including DMSO control, 2-DG (1 mM), and 2-DG (2.5 mM). (G) Flow cytometry analysis of sorted CFU-E cells into GPA^+^ cells at 24 h after 2-DG treatment. (H) The proportion of CFU-E and GPA^+^ cells were analyzed 24 h after 2-DG treatment. (I) Colony-forming ability of erythroid cells derived from DMSO control and 2-DG (2.5 mM) in CFU-E colony medium; scale bars, 50 μm. (J and K) Quantitative analysis the number and area of CFU-E colonies from 3 independent experiments. Statistical analysis is from 3 independent experiments, and the bar plot represents mean ± SD of triplicate samples. ns, not significant; ∗*p* < 0.05, ∗∗*p* < 0.01, and ∗∗∗*p* < 0.001.

The glycoprotein GPA is an essential factor for the erythroid progenitor cells to differentiate into terminal erythroid cells. To clarify the influence of glycolysis on the differentiation of erythroid progenitor cells, we treated erythroid cells with 2-DG on day 4 and detected their differentiation on day 6. The results revealed that only ∼13.2% and ∼21.1% of erythroid cells were CFU-E in the 2-DG treatment groups compared to ∼25.3% in the control group (Figures 2A and 2B). Additionally, there was a 10.6%–18.9% increase in the proportion of GPA^+^ cells compared to the control group (Figures 2C and 2D). Morphological analysis showed that 2-DG treatment resulted in a dramatic decrease in the size of erythroid cells (Figures 2E and 2F). Similarly, when sorted CFU-E cells were treated with 2-DG for 24 h, there was a decrease in the proportion of CFU-E cells and an increase in the GPA^+^ cells population compared to the control group (Figures 2G and 2H). Meanwhile, examination of the colony-forming ability of erythroid progenitor cells indicated that treatment with 2-DG led to a reduction in both number and size of cell colonies (Figures 2I–2K). These findings suggest that inhibition of glycolysis promotes erythroid differentiation while suppressing the colony forming ability of erythroid progenitor cells.

**Figure 3:**
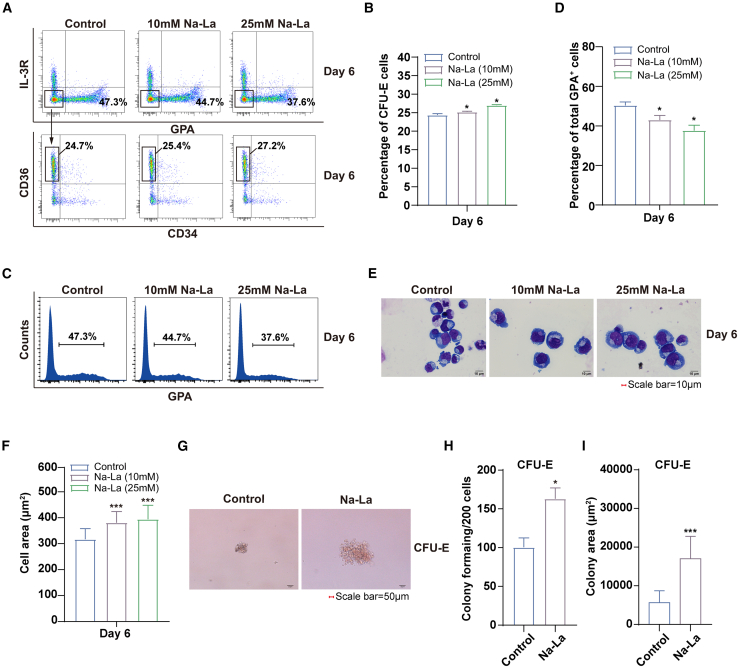
Na-La treatment delayed differentiation of early stage of human erythropoiesis (A) Flow cytometry analysis of erythroid progenitor cells at day 6, including DMSO control, Na-La (10 mM), and Na-La (25 mM). (B) Statistical results of the percentage of CFU-E cells to total cell. (C) Flow cytometry analysis of GPA in erythroid cells cultured on day 6, including DMSO control, Na-La (10 mM), and Na-La (25 mM). (D) Statistical results of the percentage of the GPA^+^ cells to total cell. (E) Representative images of DMSO control, Na-La (10 mM), and Na-La (25 mM) on day 6. (F) Quantitative analysis of cell area of day 6 erythroid cells (*n* = 100) from 3 independent experiments, including DMSO control, Na-La (10 mM), and Na-La (25 mM). (G) Colony-forming ability of erythroid cells derived from DMSO control and Na-La (25 mM) in CFU-E colony medium; scale bars, 50 μm. (H and I) Quantitative analysis the number and area of CFU-E colonies from 3 independent experiments. Statistical analysis is from 3 independent experiments, and the bar plot represents mean ± SD of triplicate samples. ns, not significant; ∗*p* < 0.05, ∗∗*p* < 0.01, and ∗∗∗*p* < 0.001.

We further treated erythroid progenitors with Na-La to explore the effect of elevated lactate on erythroid differentiation and colony formation. The results showed a significantly increased percentage of CFU-E and a decreased percentage of GPA^+^ cells in the 10 mM and 25 mM Na-La groups compared to the control group (Figures 3A–3D). Morphological analysis indicated that the size of cells increased in the Na-La-treated group compared to the control group (Figures 3E and 3F). These results suggest that elevated lactate levels delay erythroid differentiation. Furthermore, cloning forming assays demonstrated significantly increased number of colonies and larger colony size in Na-La-treated erythroid progenitor cells (Figures 3G–3I). In conclusion, adding lactate had an opposing effect on erythroid progenitor cell differentiation and colony formation as glycolysis was inhibited. Therefore, inhibition of lactate levels resulted in promoted differentiation and impaired colony formation of erythroid progenitor cells due to reduced lactate levels.

### Lactate regulates histone lactylation modifications during early stages of erythroid differentiation

**Figure 4:**
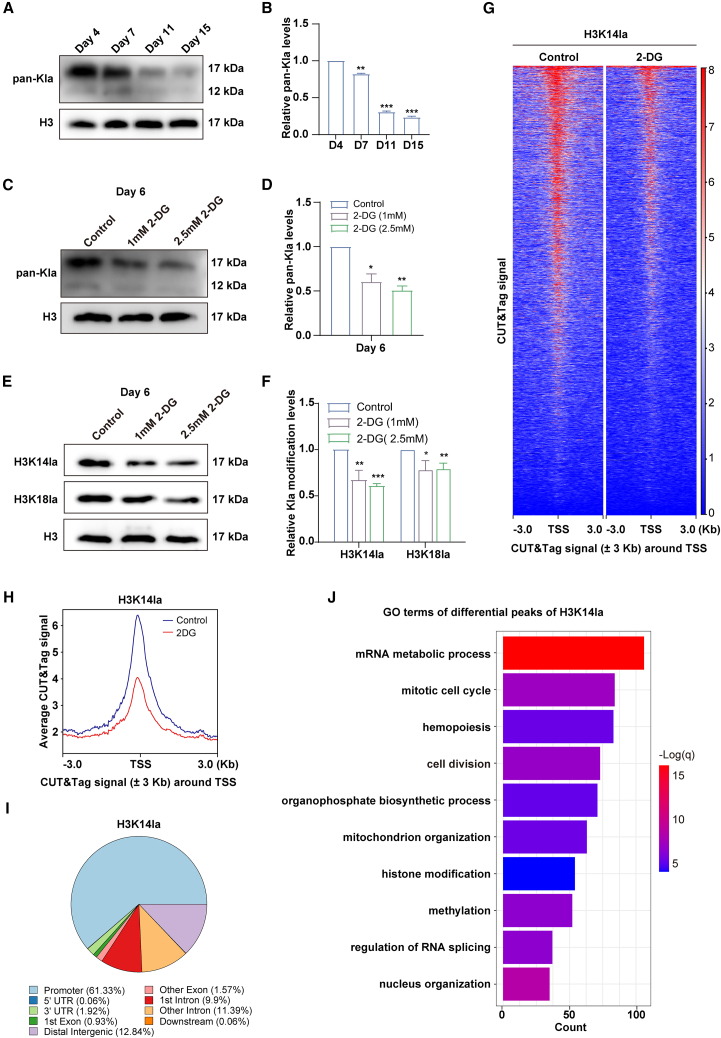
Lactate regulates histone lactylation modifications during early stages of erythroid differentiation (A) Representative western blot showing the level of histone Kla in whole cell lysates prepared from cultured erythroid cells on days 4, 7, 11, and 15. (B) Quantitative analysis of Kla from 3 independent experiments. (C) Representative western blot showing the level of histone Kla in cells, including DMSO control, 2-DG (1 mM), and 2-DG (2.5 mM). H3 was used as loading control. (D) Quantitative analysis of the relative level of pan Kla from 3 independent experiments. (E) Representative western blot showing the Kla modification level of H3K14la and H3K18la in cells, including DMSO control, 2-DG (1 mM), and 2-DG (2.5 mM). H3 was used as loading control. (F) Quantitative analysis of the relative Kla modification level of H3K14la and H3K18la from 3 independent experiments. (G) Heat maps of CUT&Tag signals around TSS for DMSO control (left) and 2.5 mM 2-DG (right). (H) Representative peak plots of CUT&Tag signals around the TSS for DMSO control (blue) and 2.5 mM 2-DG (red). (I) Pie plot of the distribution of differential CUT&Tag peaks related to gene features after 2-DG treatment. (J) GO analysis showed functional classification of H3K14la CUT&Tag peaks linked to gene promoters after 2-DG treatment. Statistical analysis is from 3 independent experiments, and the bar plot represents mean ± SD of triplicate samples. ∗*p* < 0.05, ∗∗*p* < 0.01, and ∗∗∗*p* < 0.001.

Lactate-regulated histone lactylation is a novel epigenetic modification that is involved in tumorigenesis and macrophage reprogramming by affecting gene expression.^17^^,^^25^ To explore the effect of altered lactate levels on histone lactylation modifications in erythroid progenitor cells, we collected cells at days 4,7, 11, and 15 during erythropoiesis and detected the levels of pan-lysine lactylation (Pan Kla) by western blot. The results showed that, consistent with lactate levels, erythroid progenitors exhibited significantly higher levels of Pan Kla than the erythroid terminal differentiation stage (Figures 4A and 4B). Treatment with 2-DG on day 4 of erythroid cells led to decrease expression level of Pan Kla modification, indicating that lactate levels affect erythroid progenitor development by regulating lactylation (Figures 4C and 4D). Additionally, treatment with Na-La resulted in significantly increased expression level of Pan-Kla modified proteins (Figures S3A and S3B). Subsequently, we detected the level of the histone H3K14 lactylation (H3K14la) and H3K18 lactylation (H3K18la). Our results demonstrated a decrease in the abundance of H3K14la and H3K18la in the 2-DG group compared to the control group (Figures 4E and 4F), which exhibited a similar trend to that observed for Pan Kla levels. In contrast, Na-La treatment increased the abundance of H3K18la and H3K14la (Figures S3C and S3D). Moreover, the most significant change in H3K14la abundance was observed after 2-DG or Na-La treatment (Figures 4E, 4F, S3C, and S3D). These findings suggest that alterations in lactate levels regulate the abundance of histone lactylation at the early stage of erythropoiesis.

To investigate the role of histone lactylation in gene regulation at the early erythroid progenitor stage, we performed H3K14la Cleavage Under Targets and Tagmentation (CUT&Tag) sequencing (given that H3K14la abundance changed most significantly) to explore its genome-wide distribution. Principal-component analysis and Spearman’s correlation analysis revealed high reproducibility between replicates of two groups, indicating distinct gene expression patterns of two groups (Figures S4A and S4B). After 2-DG treatment, the number of peaks for H3K14la changed dramatically, with 8,160 lost peaks, 2,799 shared peaks, and 416 gained peaks (Figure S4C). CUT&Tag signaling heatmap and profile showed that there was a dramatic reduction of H3K14la peak signal at the 3 kb region around transcriptional start site (TSS) after 2-DG treatment (Figures 4G and 4H). Genomic annotation results showed that lost CUT&Tag peaks for H3K14la were mainly enriched in promoter regions (61.33%) (Figure 4I). Based on gene ontology (GO) analysis, we found that the genes associated with lost peaks for H3K14la in promoter regions were mainly related to hemopoiesis, cell cycle, cell division, mitochondrial organization, mRNA metabolic pathway (Figure 4J), as well as nuclear organization and epigenetic modifications (histone modification and methylation), which were represented in the genes associated with the lost peaks for H3K14la. Among them, cell cycle and division have been shown to be closely related to stem cell pluripotency and differentiation,^26^ which may be associated with impaired colony formation and accelerated differentiation of CFU-E. In summary, these results show that inhibiting lactate levels lead to a direct reduction in histone lactylation levels and changes in H3K14la levels, which may be related to impaired colony forming capacity and promotion of differentiation in CFU-E.

### Gene expression regulated by histone lactylation modifications affects early stages of erythroid differentiation

**Figure 5:**
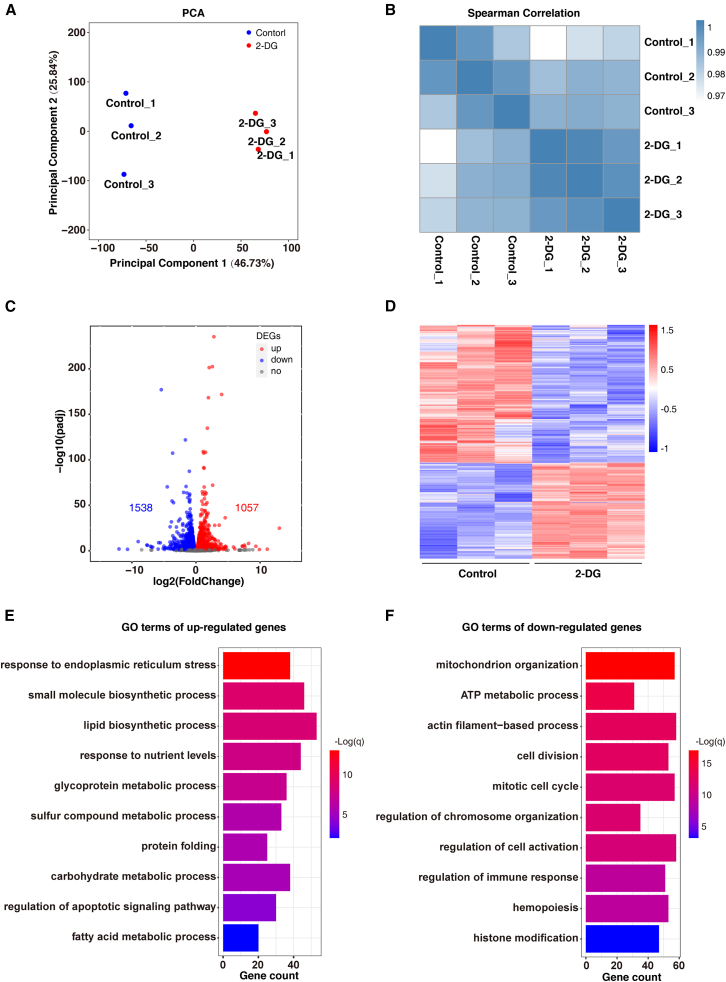
RNA-seq reveals altered gene expression patterns in CFU-E after 2-DG treatment (A) Principal-component analysis of samples representing 3 biologic replicates from CFU-E cells treated with DMSO control or 2.5 mM 2-DG. (B) Pearsons’s correlation analysis of RNA-seq. (C) Volcano map showing genes with significant difference between DMSO control and 2-DG treated group. (D) Heatmap showing expression values of differentially expressed genes (DEG) between DMSO control and 2-DG treated group. (E) The top upregulated pathways revealed by gene ontology (GO) analysis of the differentially expressed genes between DMSO control and 2-DG treated group. (F) The top downregulated pathways revealed by gene ontology (GO) analysis of the differentially expressed genes between DMSO control and 2-DG treated group.

Previous studies have found that histone lactylation modifications directly regulate gene transcription in chromatin.^17^ To examine the effect of the inhibition of histone lactylation modifications using 2-DG on the gene expression pattern of CFU-E stage cells, we sorted CFU-E cells from day 6 of erythroid differentiation and RNA sequencing (RNA-seq) was performed after a 24-h treatment with 2-DG. Principal-component analysis revealed tight clustering of transcriptomes from 2-DG treatment groups and control groups, even between biologically different replicates (Figure 5A). Differences in gene expression patterns were observed after 2-DG treatment. Pearson correlation analysis also showed a high degree of reproducibility between different biological replicates (Pearson >0.9 for 2-DG treatment and control (Figure 5B). Differential analysis results revealed that gene expression patterns changed dramatically after 2-DG treatment. A total of 2,615 differentially expressed genes (DEGs) were identified, including 1,057 upregulated genes and 1,538 downregulated genes (Figures 5C and 5D). The GO analysis results indicated that upregulated genes were enriched in various metabolic pathways, such as lipid and glycoprotein metabolism (Figure 5E). In addition, these upregulated genes were also enriched in protein folding, endoplasmic reticulum stress, and apoptosis (Figure 5E). Previous studies have demonstrated that abnormal protein synthesis and folding can induce endoplasmic reticulum stress, ultimately leading to cell apoptosis.^27^ Conversely, the downregulated genes were primarily associated with cell cycle, cell division, regulation of immune response, hemopoiesis, and chromosome organization. These findings suggest that the cell cycle observed in CFU-E cells after 2-DG treatment may be attributed to the downregulation of genes related cell cycle and division-related genes (Figure 5F).

**Figure 6:**
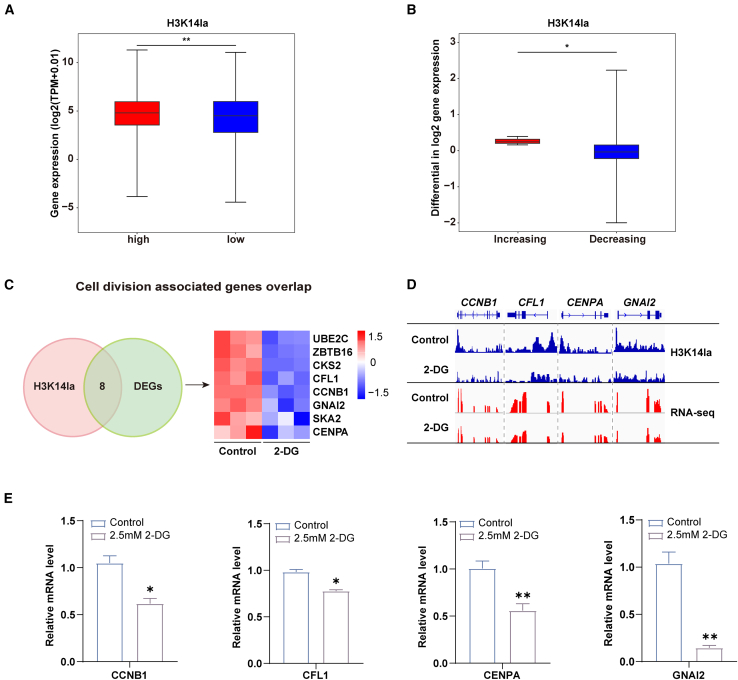
Gene expression regulated by histone lactylation modifications during early stages of erythroid differentiation (A) Heatmap analysis of the expression of genes involved in glycolysis and oxidative phosphorylation by RNA-seq during each erythroblast stage, cultured from normal human. High: higher than the median H3K14la signal of all genes. Low: lower than the median H3K14la signal of all genes. (B) Boxplots of gene expression differences of the H3K14la CUT&Tag peak located in promoter for the signal increasing and decreasing groups. (C) Veen plot and heatmap of CUT&Tag and RNA-seq showed H3K14la-enriched differential gene expression values. (D) Histone lactylation and gene expression levels of cell division associated gene locus in DMSO control and 2-DG treated group. (E) Quantitative analysis of the relative mRNA expression level of cell division associated gene from 3 independent experiments and the bar plot represents mean ± SD of triplicate samples. ∗*p* < 0.05 and ∗∗*p* < 0.01.

The previous results showed that H3K14la was predominantly distributed in the promoter region. Therefore, we analyzed the relationship between the densities of H3K14la in the promoter region and gene expression levels. We firstly categorized H3K14la peaks into low-densities and high-densities groups based on their densities within the promoter region to examine the differences in promoter-associated gene expression between the two groups. The results showed that the expression levels of promoter-associated genes with high densities of H3K14la peaks were significantly higher than those linked to promoters with low densities of H3K14la peaks (Figure 6A). Moreover, the expression of the linked genes that lost H3K14la peaks was significantly lower than the linked genes that gained H3K14la peaks after 2-DG treatment (Figure 6B). These results suggest that histone lactylation modifications play a role in regulating gene expression during erythroid differentiation. Previous results showed that elevated lactate levels did not affect cell proliferation and cycle, but the lost H3K14la peak after 2-DG treatment was enriched in the cell cycle and division pathway, and cell cycle and division-related genes were demonstrated to be closely associated with stem cell pluripotency and differentiation. Therefore, we screened 8 DEGs associated with cell cycle and division that were accompanied by reduced H3K14la abundance (Figure 6C). At the single-gene level, promoter-associated H3K14la peaks were significantly decreased after 2-DG treatment, with concomitant reductions in detectable gene expression at the *CCNB1*, *CFL1*, *CENPA*, and *GNAI2* gene locus (Figure 6D). To validate the RNA-seq and CUT&Tag data in CFU-E cells from on day 6 of erythroid differentiation, we conducted RT-qPCR analysis to confirm the decreased expression of target genes (Figure 6E). These results suggest that lactylation modifications may affect cell colony formation and differentiation of erythroid progenitors by regulating the expression of cell cycle and division-related genes. Taken together, these results suggest that altered histone lactylation modifications resulting from 2-DG treatment impact erythroid differentiation by regulating gene expression.

## Discussion

Histone modifications regulate erythroid development and lead to aberrant transcription during erythropoiesis.^28^^,^^29^^,^^30^ Recently, a new type of histone modification, known as histone lactylation, has been identified^17^^,^^31^^,^^32^; however, its role in erythropoiesis had not been previously reported. Given that early erythroid progenitor cells show active glycolysis, which produces large amounts of lactate as a substrate for histone lactylation, we explore the potential role of histone lactylation in erythropoiesis for the first time. We found that the regulation of lactate levels influences the levels of lactylation modification and is involved in modulating the binding sites of erythroid-specific transcription factor, thereby altering gene expression patterns and affecting the cell cycle and differentiation of erythroid progenitor cells during early erythropoiesis.

Lactate is a significant product of glycolysis, and previous studies have demonstrated that treatment with the glycolysis inhibitor 2-DG decreases intracellular lactate levels, while supplementation with exogenous lactate-Na-La increases intracellular lactate levels.^22^ To investigate how altered intracellular lactate levels affect early erythroid progenitors, we examined their early differentiation and capacity for erythroid development following exposure to 2-DG or Na-La on day 4 of erythropoietin (EPO)-induced HSC. Our research revealed that inhibition glycolysis using 2-DG resulted in decreased intracellular lactate levels, inhibited proliferation, impaired colony formation ability, and accelerated differentiation of erythroid progenitor cells. Conversely, supplementing with exogenous lactate enhanced colony formation ability and arrested differentiation in these cells. Previous studies have found that LDHA deficiency reduces lactate production and impairs the cloning ability of early erythroid progenitors, which is consistent with our findings.^33^ Similarly, in malignant melanoma cells, reduced levels of lactate and lactylation were found to diminish the clonogenic capacity of melanoma cells.^22^ The ability of cells to colony is closely related to their proliferative potential.^34^^,^^35^ Taken together, these results suggest that inhibiting lactate levels decreases the proliferative capacity of erythroid early progenitor cells, while increasing lactate levels promotes their proliferation.

Furthermore, previous studies have also supported our results by showing that 2-DG analog treatment enhances the magnitude and kinetics of HSC erythroid commitment induced by EPO.^36^ Our work further reveals lactate, as a downstream metabolite of glycolysis under 2-DG-mediated inhibition, regulates erythroid differentiation through histone lactylation modifications.

The proliferative capacity of early erythroid progenitors directly affects their self-renewal capacity, and maintaining a balance between self-renewal and differentiation is crucial for erythropoiesis.^37^^,^^38^ In this study, we found that the reducing lactate levels led to slower proliferation and promoted differentiation of erythroid progenitor cells. This may be attributed to the decrease in self-renewal capacity caused by reduced lactate levels disrupting the balance between self-renewal and differentiation. Therefore, it can be inferred that maintaining appropriate levels of lactate is essential for preserving this delicate equilibrium in early progenitor cells.

In previous studies, lactate was widely recognized as an energy source and metabolic by-product. Recently, more studies have shown that lactate-regulated histone lactylation is a new epigenetic modification and lactate-derived lysine lactylation in histones is a novel histone mark; 28 lactylation sites have been identified on core histones, including H3K4, H3K18, H3K9, and H3K14.^17^ In the present study, we found that both 2-DG and Na-La treatments significantly altered the abundance of H3K14la during early stages of erythroid development. We further explored the binding status of H3K14la-marked genomic regions to chromatin DNA and found that H3K14la-marked locus located in CFU-E stage were mainly enriched in promoter regions and that genes marked by lost H3K14la peaks after 2-DG treatment were associated hematopoietic process, cell cycle and division, mitochondrial reorganization, and mRNA metabolism pathways. This suggests that histone lactylation modifications may regulate the expression of genes related to self-renewal and differentiation of erythroid progenitor cells and thus affect erythroid development.

Previous studies have shown that lactate affects gene expression by regulating histone lactylation modifications. Increased histone lactylation in promoter regions has been proven to induce the expression of homeostatic genes, including Arg1, during M1 macrophage polarization caused by infection.^17^ Histone lactylation promoted the transcription of Rubicon Like Autophagy Enhancer (RUBCNL)/Pacer, facilitating autophagosome maturation.^39^ Histone lactylation promotes oncogenesis by enhancing the expression of m6A reader YTHDF2 in ocular melanoma.^40^ Additionally, hexokinase 2-mediated gene expression via histone lactylation is crucial for hepatic stellate cells and liver fibrosis.^41^ Our study revealed significant changes in gene expression profile during the early erythroid stages of 2-DG-treated cells. The differentially upregulated genes were found to be involved in lipid metabolism, which are essential for stemness maintenance, self-renewal, and differentiation of stem cells^42^ as well as erythropoiesis.^43^ Conversely, the differentially downregulated genes are involved in cell cycle, cell division, immune response, and hematopoiesis. In addition, our analysis by CUT&Tag sequencing indicated that H3K14la-associated peaks located in the promoter region were mainly linked to hematopoietic process, cell cycle and division, mitochondrial reorganization, and mRNA metabolism pathways. These findings align with biological functions of DEGs and suggest a role for lactylation modification in transcriptional regulation during early stages of erythroid differentiation.

Previous results showed that proliferation and cell cycle of early erythroid progenitors were impaired by 2-DG treatment, but increased lactate levels did not affect cell proliferation and cell cycle. CUT&Tag results showed that lost H3K14la peaks by 2-DG treatment were enriched in the cell cycle and division pathways, and that cell cycle and division-related genes proved to be closely associated with stem cell pluripotency and differentiation.^26^ Further analysis revealed that the levels of lactylation modifications with 8 promoter regions of cell cycle and division-related differential genes were altered after 2-DG treatment. Specifically at the single gene level, the promoter-associated H3K14la peak was significantly reduced after 2-DG treatment, as was detectable gene expression at the *CCNB1*, *CFL1*, *CENPA*, and *GNAI2* gene loci. The *CCNB1* gene encodes cyclin B1, whose abundance is required for embryonic stem cells (ESCs) self-renewal and pluripotency and plays an important role in cell cycle progression in ESCs.^44^ *CFL1* is a protein that regulates actin polymerization and depolymerization, and downregulation of its expression promotes differentiation of bovine primitive myoblasts.^45^ In addition, *CFL1* acts as a dynamic regulator of neural stem cell fate decisions. Specifically, over-expression of *CFL1* significantly inhibits cell differentiation and neurite outgrowth, whereas inhibition of intracellular CFL1 phosphorylation was significantly promoted.^46^ Decreased phosphorylation of CFL1 has been shown to reduce K562 proliferation and increase the expression of erythroid differentiation markers GPA and hemoglobin in TF-1 cells.^47^ Centromere protein A encoded by the *CENPA* gene plays an irreplaceable role in kinetochore assembly, chromosome segregation, and cell division. Latest study shows that CENPA promotes stemness in glioma stem cells.^48^ The *GNAI2* gene encodes G protein subunit alpha i2; inactivation of the IL-8/GNAI2 pathways inhibits CD34^+^ cell proliferation and colony formation.^49^

### Limitations of the study

Our study reveals that histone lactylation modifications during erythropoiesis affect cell differentiation of erythroid progenitors by regulating gene expression. These findings provide new insights into the role of lactate in regulating erythropoiesis. However, we have not yet demonstrated the mechanism by which histone lactylation regulated gene expression. Thus, further research will be needed to elucidate this. While our study demonstrates that 2-DG reduces intracellular lactate levels and links this effect to histone lactylation-mediated gene regulation, we acknowledge that 2-DG may also perturb the abundance of other metabolites (e.g., glycolytic intermediates, nucleotides, and redox cofactors) through its inhibition of hexokinase. Nevertheless, 2-DG remains a widely used and well-characterized pharmacological tool to suppress glycolysis and deplete intracellular lactate in experimental systems.^50^ Future studies employing metabolomic profiling or isotope tracing could further dissect the broader metabolic consequences of 2-DG treatment in this context.

## Resource availability

### Lead contact

Further information and requests should be directed to the lead contact, Xiuyun Wu (wuxy@zzu.edu.cn).

### Materials availability

This study did not generate new unique reagents.

### Data and code availability

• RNA-seq and CUT&Tag datasets for erythroid cells induced by human umbilical cord blood-derived CD34^+^ HSCs generated during the current study are available at the GEO under the accession codes GEO: GSE274169 (RNA-seq) and GEO: GSE274165 (CUT&Tag). Published RNA-seq datasets of distinct stages of erythroid cells induced by human umbilical cord blood-derived CD34^+^ HSCs from studies by An et al.^20^ and Li et al.^21^ are available at the GEO under accession GEO: GSE61566 and GEO: GSE53983. There are no restrictions on data availability.
• This paper does not report original code.
• Any additional information required to reanalyze the data reported in this paper is available from the lead contact upon request.

## Acknowledgments

This work was supported by grants from the 10.13039/501100001809National Natural Science Foundation of China (82300134, 82170116, and 81870094).

## Author contributions

Q.Y., writing – original draft, visualization, validation, software, project administration, methodology, investigation, formal analysis, data curation, and conceptualization. H.Z., writing – original draft, visualization, software, methodology, investigation, formal analysis, and data curation. Y.H., writing – original draft, visualization, validation, formal analysis, and data curation. S.G., methodology, data curation, and validation. L.C., supervision and project administration. F.X., writing – review & editing, supervision, and funding acquisition. X.W., writing – review & editing, supervision, and conceptualization.

## Declaration of interests

The authors declare no competing interests.

## STAR★Methods

### Key resources table

**Table:** 

REAGENT or RESOURCE	SOURCE	IDENTIFIER
**Antibodies**		

Rabbit polyclonal anti-Pan Kla	PTM BIO	Cat# PTM-1401;RRID:AB_2868521
Rabbit anti-H3K18la	PTM BIO	Cat# PTM-1406RM;RRID:AB_2909438
Rabbit anti-H3K14la	PTM BIO	Cat# PTM-1414RM;RRID:AB_3076697
Rabbit anti-H3	PTM BIO	Cat# PTM-1001RM;RRID:AB_3676032
APC-conjugated CD235a (GPA)	BD Pharmingen	Cat# 551336;RRID:AB_398499
PE-conjugated CD235a (GPA)	BD Pharmingen	Cat# 561051;RRID:AB_10563407
PE-conjugated CD34	BD Pharmingen	Cat# 555822;RRID:AB_396151
APC-conjugated CD49d (α4 integrin)	MACS	Cat# 130-124-797;RRID:AB_2802060
FITC-conjugated CD36-fluoresce	BD Pharmingen	Cat# 555454;RRID:AB_2291112
PE-cy7- conjugated CD123 (IL-3R)	Invitrogen	Cat# 25-1239-42
APC-conjugated annexin V	eBioscience	Cat# 88-8103-74
FITC-conjugated annexin V	Biolegend	Cat# 640945
7-AAD	eBioscience	Cat# 00-6993-50
Hoechst33342	Solarbio	Cat# C0031
HRP conjugated Goat Anti-Rabbit IgG (H + L)	Servicebio	Cat# GB23303;RRID:AB_2811189

**Biological samples**		

Cord blood	YINFENG BIOLOGICAL GROUP	N/A

**Chemicals, peptides, and recombinant proteins**		

2-Deoxy-D-glucose (2-DG)	MCE	Cat# 154-17-6
L-Lactate (Na-La)	Sigma	Cat# 867-56-1
Erythropoietin (EPO)	STEMCELL	Cat# 04330
May-Grünwald	Sigma	Cat# MG500
MethoCult H4434 classic medium	STEMCELL	Cat# 04434
Giemsa solution	Sigma	Cat# 51811-82-6
RIPA lysis buffer	Thermo Fisher Scientific	Cat# 89900
Proteinase inhibitor Cocktail	Sigma	Cat# P8340

**Critical commercial assays**		

Click-iT™ EdU Alexa Fluor™ 647	Thermo Fisher Scientific	Cat# C10119
L and D lactate assay kit	AAT Bioquest	Cat# 13815
RNA extract kits	Tiangen Biotech	Cat# 74104
HiFi-MMLV cDNA kit	CWBIO	Cat# CW0744M
SYBR™ Select Master Mix	Thermo Fisher Scientific	Cat# 4472903
BCA protein assay kit	PPLYGEN	Cat# P1511-5
Glycolysis/OXPHOS Assay kit	DOJINDO	Cat# G270
HyperactiveTM *In-Situ* CUT&Tag Library Prep Kit	Vazyme	Cat# TD901

**Deposited data**		

RNA-seq	This paper	GEO: GSE274169
RNA-seq: Distinct stages of erythropoiesis	Li et al.^21^; An et al.^20^	GEO: GSE61566, GSE53983
CUT&Tag: H3K14la	This paper	GEO: GSE274165
CUT&RUN: KLF1	Dong Li et al. 2023^51^	GEO: GSE183989
Human reference genome NCBI build 38, GRCh38	Genome Reference Consortium	http://www.ncbi.nlm.nih.gov/projects/genome/assembly/grc/human/

**Oligonucleotides**		

qPCR primers for CCNB1: Forward: TGTTGGTTTCTGCTGGGTGT Reverse: AGCTGTTCTTGGCCTCAGTC	This paper	N/A
qPCR primers for CFL1: Forward: GGTGCCCTCTCCTTTTCGTT Reverse: TTGACAAAGGTGGCGTAGGG	This paper	N/A
qPCR primers for CKS2: Forward: TCTTCGCGCTCTCGTTTCAT Reverse: TGGACACCAAGTCTCCTCCA	This paper	N/A
qPCR primers for GNAI2: Forward: GTTAGCCGCTGTCCATTGCT Reverse: CATTCCTCCTCGGAGTAGCC	This paper	N/A
qPCR primers for β-actin: Forward: CCTGGCACCCAGCACAAT Reverse: GCTGATCCACATCTGCTGGAA	This paper	N/A

**Software and algorithms**		

Python version 2.7	Python Software Foundation	https://www.python.org
HISAT2 version 2.2.1	Kim et al.^52^	http://daehwankimlab.github.io/hisat2/
StringTie version 2.1.7	Pertea et al.^53^	https://ccb.jhu.edu/software/stringtie/
DESeq2 version 1.38.3	Anders and Huber, 2010^54^	https://bioconductor.org/packages/3.0/bioc/html/DESeq2.html
Trim Galore version 0.6.6	Kechin et al.^55^	https://github.com/FelixKrueger/TrimGalore
Bowtie2 version 2.3.5.1	Langmead and Salzberg^56^	https://sourceforge.net/projects/bowtie-bio/files/bowtie2/2.3.5.1/
DiffBind version 3.8.4	Ross-Innes et al.^57^	https://bioconductor.org/packages/release/bioc/html/DiffBind.html
Deeptools version 2.5.7	Ramírez et al.^58^	https://deeptools.readthedocs.io/en/develop/content/installation.html
MACS2 version 2.1.1	Zhang et al.^59^	https://pypi.org/project/MACS2/
ChIPseeker version 1.34.0	Yu et al.^60^	https://www.bioconductor.org/packages/3.9/bioc/html/ChIPseeker.html
Homer version 4.11	Heinz et al.^61^	http://homer.ucsd.edu/homer/

**Other**		

Pannoramic MIDI	3DHISTECH	https://www.3dhistech.com/research/pannoramic-digital-slide-scanners/pannoramic-midi/
SpectraMax i3	Molecular Devices	https://www.moleculardevices.com/sites/default/files/en/assets/brochures/br/spectramax-i3-multi-mode-platform.pdf
Axio Imager.A2	Carl Zeiss Microscopy GmbH	https://www.zeiss.com.cn/microscopy/products/light-microscopes/axio-imager-2-for-biology.html
LKB 2088 Ultratome V	Bromma	N/A
120-Kv H-7700 Hitachi transmission electron microscope	Hitachi High-Tech	N/A
Confocal laser scanning microscope LSM780	Zeiss	https://www.zeiss.com/content/dam/Microscopy/Downloads/Pdf/FAQs/zen2010-lsm780_basic_fcs_experiments.pdf
NanoDrop™ 2000/2000c Spectrophotometers	Thermo Scientific	Cat#ND-2000C
LightCycler® 480 System	Roche Life Science	https://lifescience.roche.com/global/en/article-listing/article/lightcycler-480-system-technology.html
BD LSRFortessa™ flow cytometry	BD Biosciences	https://www.bdbiosciences.com/en-us/products/instruments/flow-cytometers/research-cell-analyzers/bd-lsrfortessa

### Experimental model and study participant details

#### Acquisition and culture of CD34^+^ cells

CD34^+^ cells were purified from cord blood purchased from YINFENG BIOLOGICAL GROUP using magnetic selective beads. The details of cells culture have been described previously.^4^ The details were as follows: The day of CD34^+^ cell harvest was recorded as day 0. The cell culture procedure consisted of 3 phases. The basic culture medium consisted of Iscove’s modified Dulbecco’s medium, 2% human peripheral blood plasma, 3% human AB serum, 200 mg/mL human holo-transferrin, 3 IU/mL heparin and 10 mg/mL insulin. In the first phase (day 0 to day 6), CD34^+^ cells were cultured at a concentration of 10^5^/mL in the presence of 10 ng/mL stem cell factor, 1 ng/mL IL-3 and 3 IU/mL erythropoietin. In the second phase (day 7 to day 11), IL-3 does not need to be added to the culture medium. In the third phase, which lasted until day 21, the cell concentration was adjusted to 10^6^/mL on day 11 and to 5×10^6^/mL on day 15, the medium for this phase was the base medium plus 3 IU/mL erythropoietin, and the concentration of transferrin was adjusted to 1 mg/mL. Fresh medium was replaced on days 4 of differentiation to prevent metabolite accumulation. During replacement, old medium was aspirated, and pre-warmed fresh differentiation medium (containing the same concentrations of 2-DG or other reagents) was added to maintain experimental consistency.

#### Ethical declaration

This study involving human participants followed the principles outlined in the Declaration of Helsinki and was approved by the Ethics Committee of the Zhengzhou University (Ethics Approval ZZUIRB2023-273).

### Method details

#### Antibodies and reagents

The antibodies used for western blotting were rabbit anti-human Rabbit polyclonal anti-Pan Kla (Cat# PTM-1401, PTM BIO), rabbit anti-H3K18la (Cat# PTM-1406RM, PTM BIO), rabbit anti-H3K14la (Cat# PTM-1414RM, PTM BIO), rabbit anti-H3 (Cat# PTM-1001RM, PTM BIO), HRP conjugated Goat Anti-Rabbit IgG (Cat# GB23303, Servicebio). Commercial antibodies used for flow cytometry were as follows: APC-conjugated CD235a (GPA) (Cat# 551336, BD), PE-conjugated CD235a (GPA) (Cat# 8065789, BD), PE-conjugated CD34 (Cat# 555822, BD), APC-conjugated CD49d (α4 integrin) (Cat# 130-124-229, MACS), FITC-conjugated CD36-fluoresce (Cat# 555454, BD), PE-cy7- conjugated CD123 (IL-3R) (Cat# 25-1239-42, invitrogen) APC-conjugated annexin V (Cat# 88-8103-74, eBioscience), FITC-conjugated annexin V (Cat# 640945, Biolegend) Hoechst33342 (Cat# C0031, Solarbio), and 7AAD (Cat# 00-6993-50, eBioscience).^2^

#### Drug treatment

The drugs for cell treatment were as follows. 2-DG (Cat# 154-17-6) was purchased from MCE and it was added into cell culture at a final concentration of 1 and 2.5 mM. Na-La (Cat# 867-56-1) was purchased from Sigma and it was added into cell culture at a final concentration of 10 and 25 mM.

#### Glycolysis/OXPHOS assay

On day 6 of erythroid differentiation, control and treat cells were prepared in 96-well plate (5×10^4^ cells per well). Then the lactate in the supernatant and the ATP in the cells were measured according to the instructions of the Kit (Glycolysis/OXPHOS Assay Kit, Cat# G270, DOJINDO Laboratories, Kumamoto, Japan).

#### Flow cytometry analysis and fluorescence-activated cell sorting of erythroblasts

Early erythroid differentiation was monitored by analyzing the surface expression of glycophorin A (GPA), CD36, CD34, IL-3R as previously described.^21^ Stained cells were analyzed on a BD LSRFortessa flow cytometer and data analyses were performed using FCS Express 6 or Flow Jo software package. Erythroid cells at distinct differentiation stage were sorted using a MOFLO high-speed cell sorter (Beckman-Coulter) as previously described.^2^^,^^62^^,^^63^ Sorted CFU-E for RNA-seq and CUT&Tag, as described previously.^62^

#### Cell cycle analysis

EdU kit (Cat# C10119, Thermo Fisher Scientific) was used for cell cycle measurement according to the manufacturer’s protocol. In brief, 1 × 10^6^ cells were incubated with 10 μM EdU for 2 h at 37°C. After incubation, the cells were harvested and washed with 3 mL of 1% BSA in PBS. Cells were then fixed, permeabilized, and stained with EdU detection cocktail as well as 7AAD. The staining of EdU and 7AAD was analyzed by flow cytometry. Data were collected and analyzed using FlowJ, and the data are expressed as EdU fluorescence intensity versus 7AAD.^64^

#### Colony-forming assay

Cultured cells were plated in triplicate at a density of 200 cells in 1 mL of MethoCult H4434 classic medium (Cat# 04434, STEMCELL) or in 1mL of MethoCult H4330 medium with EPO only (Cat# 04330, STEMCELL). The cells were incubated at 37°C in a humidified atmosphere with 5% CO_2_. The BFU-E and CFU-E colonies were defined according to previously described criteria.^21^

#### Cytospin assay

Cells (0.1 × 10^6^) were suspended in 200μL PBS and then adhered to a slide by Cytospin centrifugation. Slides were then stained with May-Grünwald (Cat# MG500, Sigma) solution for 5 min. Then slides were washed with 40mMtris buffer (pH 7.2) for 90s, the slides were immediately stained with Giemsa solution (Cat# 51811-82-6, Sigma) for 15min. The images were taken using a standard light microscope (Axio Imager.A2, Carl Zeiss Microscopy GmbH, Jena).

#### RNA extraction and quantitative reverse transcription-PCR assays

RNA was extracted from cell cultures using RNA extract kits (Cat# 74104, Qiagen) according to the manufacturer’s instructions. Reverse transcription was performed using a HiFi-MMLV cDNA Kit (Cat# CW0744M, CWBIO). Quantitative reverse transcription PCR (qRT-PCR) assays were completed using SYBR Select Master Mix (Cat# 4472903, Thermo Fisher Scientific). Quantitative reverse transcription-PCR was carried out on a PCR platform (LightCycler 480, Roche Life Science) using a master mix according to the manufacturer’s instructions. Relative expression levels were normalized to that of β-actin. Detailed protocols for the RNA extraction, reverse transcription, primer design, and quantitative reverse transcription-PCR have been described previously.^21^

#### Measurement of intracellular lactate levels

Prior to lysis, cells were washed three times with ice-cold PBS to remove extracellular lactate from the culture medium. Then added ice-cold PBS for sonication and the supernatant was centrifuged for detection. The CFCS was used for lactate quantification using a commercial Amplite colorimetric L and D lactate Assay Kit (Cat# 13815, AAT Bioquest), according to the protocol provided by the manufacturer.

#### Western blotting

Total cell lysates were prepared using RIPA buffer (Cat# 89900, Thermo Fisher Scientific) in the presence of proteinase inhibitor Cocktail (Cat# P8340, Sigma). Protein concentration was measured using a BCA protein assay kit (Cat# P1511-5, PPLYGEN). All procedures were performed as described previously.^62^^,^^64^

#### Histone extraction

Histones from cells were extracted using a standard acid-extraction protocol.^65^ In brief, cells were collected and resuspended in lysis buffer (10 mM Tris-HCl, 1 mM KCl, 1.5 mM MgCl_2_, and 1 mM DTT, pH 8.0) containing protease inhibitors for nuclei extraction. The nuclei were then resuspended in acid solutions (0.2 M H_2_SO_4_), and histones were extracted over night at 4°C, followed by centrifugation at 16,000 g for 10 min at 4°C. The supernatants were collected, and the histone pellet was precipitated with 35% (final concentration) trichloroacetic acid on ice. After washing and drying, the histone pellet was dissolved in H_2_O and prepared for western blotting analysis.

#### CUT&Tag

Cells were harvested, counted and centrifuged for 3 min at 600×g at room temperature. 1×10^5^ cells were washed twice in 500μL wash buffer by gentle pipetting. Concanavalin A coated magnetic beads were prepared as the kit manual described, 10 μL of activated beads were added per sample and incubated at RT for 10 min. According to the kit instructions, cells were sequentially incubated with ConA Beads, primary antibody (anti-H3K14la antibody, secondary antibody (Goat anti-Rabbit IgG) and Hyperactive PG-TN5/PA-TN5 Transposon and then fragmented. The fragmented DNA was extracted from the samples and amplified by PCR. CUT&Tag libraries were constructed using HyperactiveTM *In-Situ* CUT&Tag Library Prep Kit (TD901, Vazyme) and sequenced on an Illumina NovaSeq platform, and 150-bp paired-end reads were generated.

Trim Galore version 0.6.6 was used to remove adapter and low-quality reads.^55^ Align paired-end reads using Bowtie2 version 2.3.5.1^56^ with options: “--end-to-end --very-sensitive”. Peak calling uses MACS2 version 2.1.1^59^ with threshold: q < 0.01, and the differential binding sites of H3K14la were calculated using the DiffBind version 3.8.4^57^ with the parameter log2foldchange ≥1 and FDR <0.05 (https://www.cruk.cam.ac.uk/core-facilities/bioinformatics-core/software/diffbind.html). Scatterplots, correlation plots, and heatmaps are displayed using deepTools version 2.5.7.^58^ Annotation of peaks is performed using an R package ChIPseeker version 1.34.1^60^ (https://www.cruk.cam.ac.uk/core-facilities/bioinformatics-core/software/ChIPseeker.html). The CUT&Tag peak/region <1000bp from the nearest TSS was defined as the promoter. Hypergeometric Optimization of Motif Enrichment version 4.11^61^ (http://homer.ucsd.edu/homer/) was used to search for the binding motif.

#### RNA-seq

Experiment procedure before sequencing: 2 million cells were collected and washed twice with PBS. RNA was extracted and used as cDNA input, cDNA library was prepared by Illumina Truseq DNA library kit, and sequenced on Illumina NovaSeq 6000.

The original data were filtered and compared with the reference genome, which can be downloaded from the Ensemble database (http://www.ensembl.org/index.html,human genome build GRCh38.87). Using HISAT2 version 2.2.1^52^ (http://daehwankimlab.github.io/hisat2/) to read the counts for each gene in each sample and gene expression was assessed by StringTie^53^ version 2.1.7 (https://ccb.jhu.edu/software/stringtie/). TPM (Transcripts Per Kilobase of exon model per Million mapped reads) was calculated to evaluate the expression level. DEGs were defined by p.adjust <0.05 and fold change >1.2 using the DESeq2 version 1.38.3^57^ (https://bioconductor.org/packages/release/bioc/html/DESeq2.html). GO enrichment analysis and visualization of DEGs was performed using the Metascape (www.metascape.org/) online tool with q < 0.05.

### Quantification and statistical analysis

Statistical analysis was performed using Prism software version 8.0 (GraphPad). Data were assessed for normal distribution and plotted in the figures as mean ± standard deviation (S.D.). Differences between two treatment groups were assessed using two-tailed, unpaired Student’s t test with Welch’s correction. Significant differences emerging from the above tests are indicated in the figures by ∗*p* < 0.05, ∗∗*p* < 0.01, ∗∗∗*p* < 0.001. Notable non-significant differences are indicated in the figures by ‘‘ns’’. Individual statistical analyses performed can be found in the figure legends.

## Supplemental information

Document S1. Figures S1–S4

Data S1. Analysis of Glycolysis/OXPHOS Metabolic Dynamics during Human Erythropoiesis
