# Supplementary material for: Lactate regulates cell differentiation of erythroid progenitor cells via histone lactylation modification
Source: iScience. 2025 Jun 9;28(7):112842. doi: 10.1016/j.isci.2025.112842 (PMC12221709; doi:10.1016/j.isci.2025.112842)
Supplement: Document S1. Figures S1–S4 [file mmc1.pdf]

iScience, Volume *28*

Supplemental information

Lactate regulates cell differentiation of erythroid progenitor cells via histone lactylation modification

Qianqian Yang, Hengchao Zhang, Yan Hou, Shaoyang Gu, Lixiang Chen, Fumin Xue, and Xiuyun Wu

Supplemental information

Figure S1

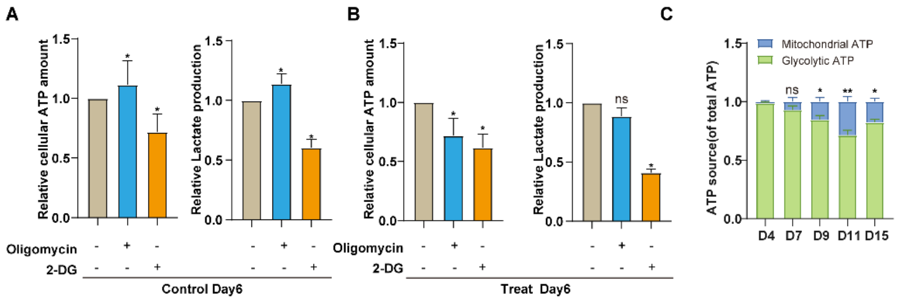

Figure S2

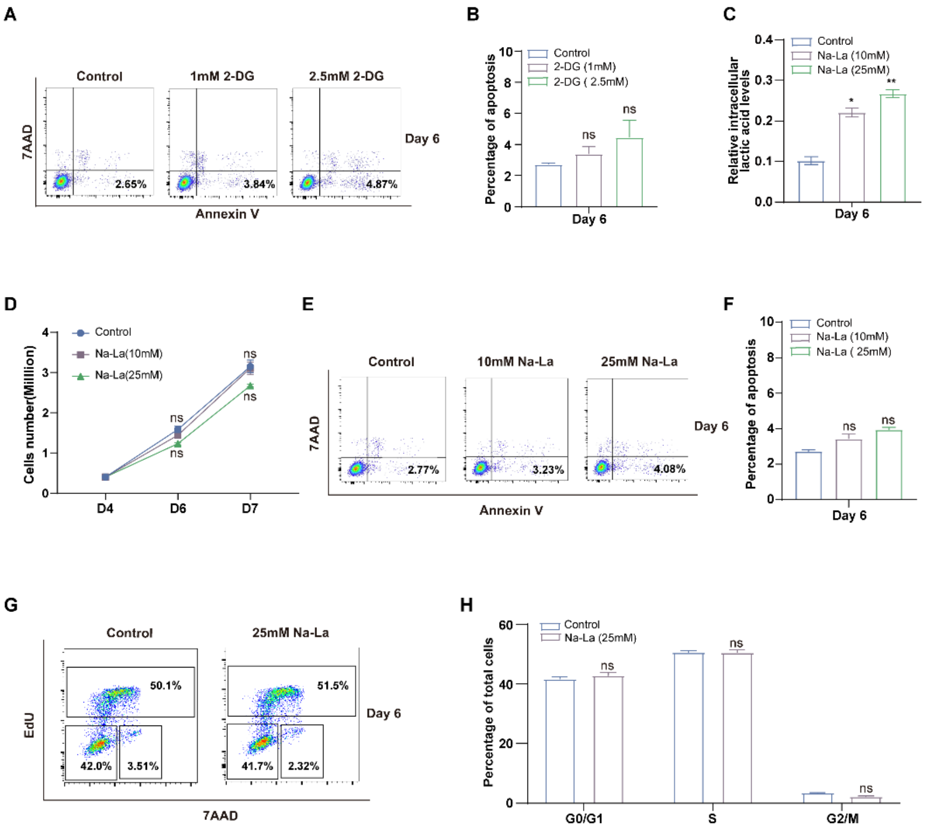
A
Control
1mM 2-DG
2.5mM 2-DG
7AAD
Day 6
2.65%
3.84%
4.87%
Annexin V
B
Percentage of apoptosis
Control
2-DG (1mM)
2-DG (2.5mM)
ns
ns
Day 6
C
Relative intracellular lactic acid levels
Control
Na-La (10mM)
Na-La (25mM)
*
**
Day 6
D
Cells number(Million)
Control
Na-La(10mM)
Na-La(25mM)
ns
ns
ns
ns
D4
D6
D7
E
Control
10mM Na-La
25mM Na-La
7AAD
Day 6
2.77%
3.23%
4.08%
Annexin V
F
Percentage of apoptosis
Control
Na-La (10mM)
Na-La (25mM)
ns
ns
Day 6
G
Control
25mM Na-La
EdU
50.1%
51.5%
Day 6
42.0%
3.51%
41.7%
2.32%
7AAD
H
Percentage of total cells
Control
Na-La (25mM)
ns
ns
ns
G0/G1
S
G2/M

Figure S3

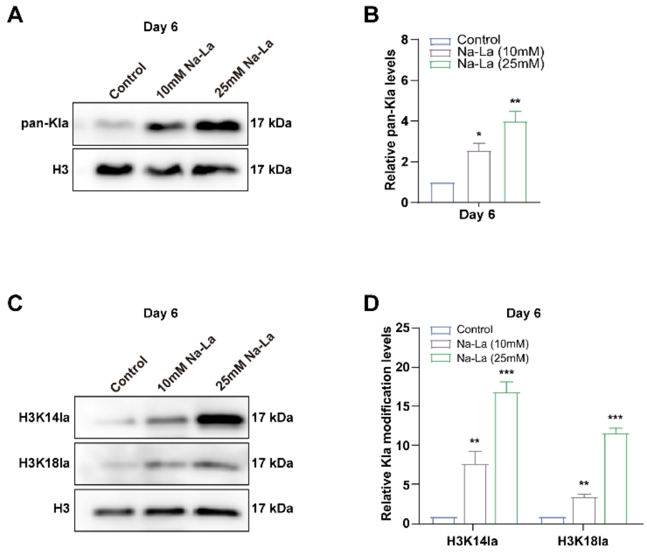

A
Day 6
Control
10mM Na-La
25mM Na-La
pan-Kla
H3
17 kDa
B
Control
Na-La (10mM)
Na-La (25mM)
Relative pan-Kla levels
Day 6
C
Day 6
H3K14la
H3K18la
H3
17 kDa
D
Day 6
Relative Kla modification levels
H3K14la
H3K18la

Figure S4

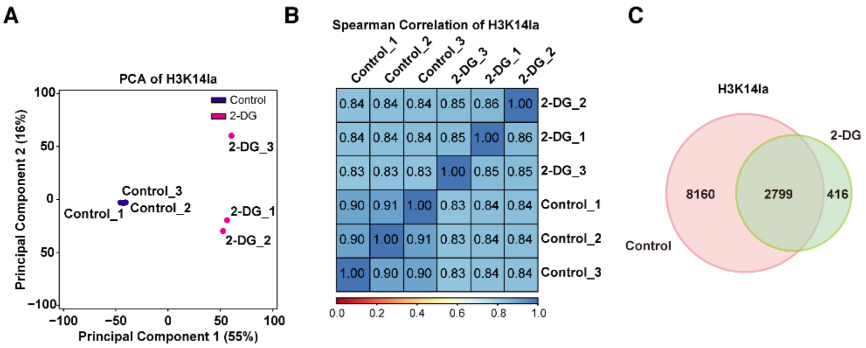
A
PCA of H3K14la
Control
2-DG
2-DG_3
Control_3
Control_1
Control_2
2-DG_1
2-DG_2
Principal Component 2 (16%)
Principal Component 1 (55%)
B
Spearman Correlation of H3K14la
Control_1
Control_2
Control_3
2-DG_3
2-DG_1
2-DG_2
0.84 0.84 0.84 0.85 0.86 1.00 2-DG_2
0.84 0.84 0.84 0.85 1.00 0.86 2-DG_1
0.83 0.83 0.83 1.00 0.85 0.85 2-DG_3
0.90 0.91 1.00 0.83 0.84 0.84 Control_1
0.90 1.00 0.91 0.83 0.84 0.84 Control_2
1.00 0.90 0.90 0.83 0.84 0.84 Control_3
0.0 0.2 0.4 0.6 0.8 1.0
C
H3K14la
2-DG
8160
2799
416
Control
