# Supplementary figures and images for: Lactate regulates cell differentiation of erythroid progenitor cells via histone lactylation modification
Source: iScience. 2025 Jun 9;28(7):112842. doi: 10.1016/j.isci.2025.112842 (PMC12221709; doi:10.1016/j.isci.2025.112842)

Figure 4A

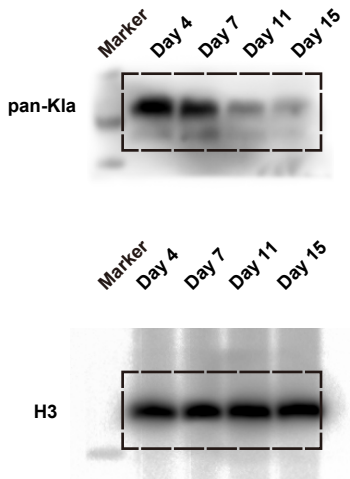

Figure 4B

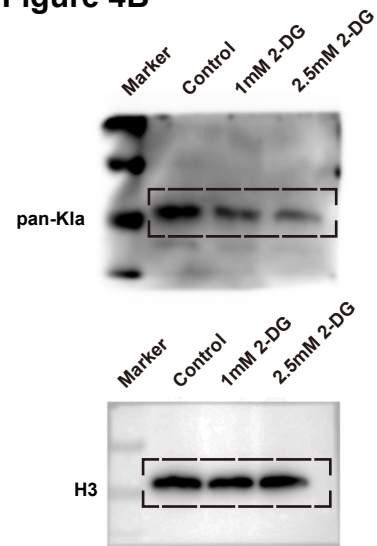

Figure 4D

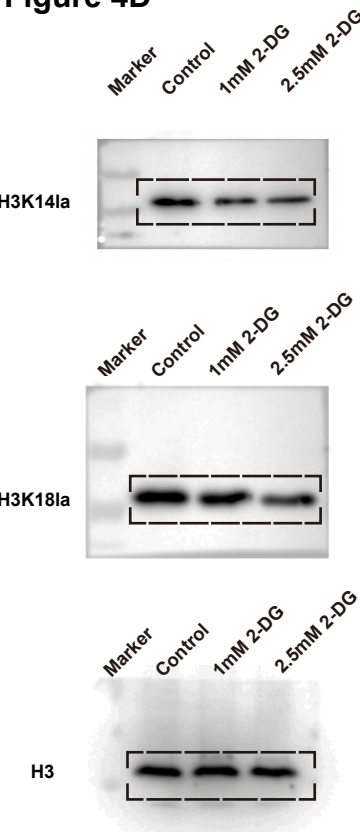

Figure S2A

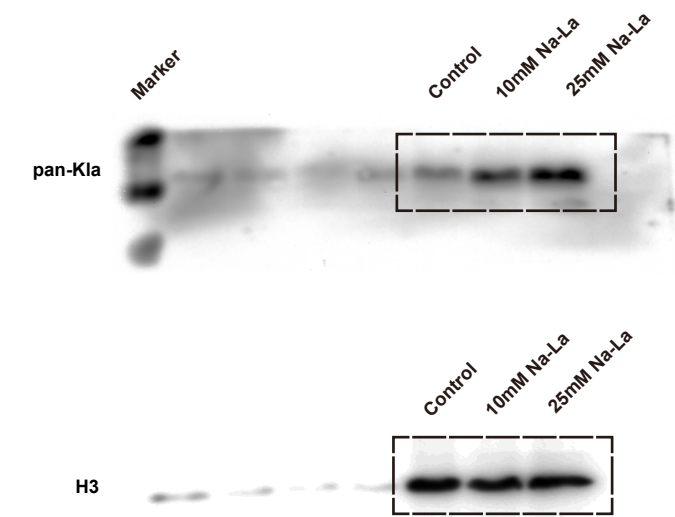

Figure S2C

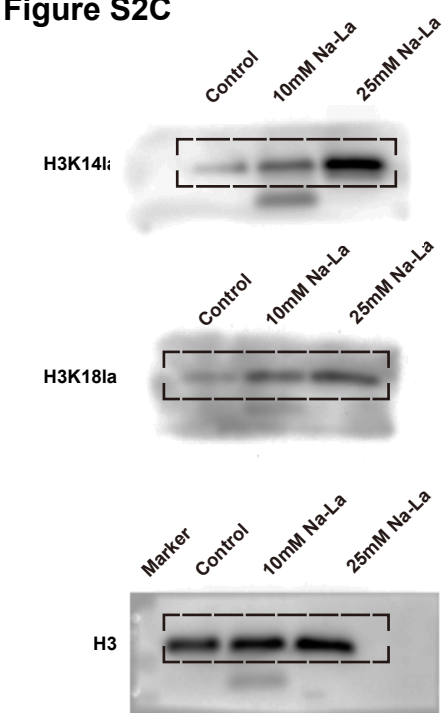

Supplement: Data S1. Analysis of Glycolysis/OXPHOS Metabolic Dynamics during Human Erythropoiesis [file mmc2.pdf]
